# Exploring the Impact of Cardiac Rehabilitation Programs on Health-Related Quality of Life and Physiological Outcomes in Patients Post Coronary Artery Bypass Grafts: A Systematic Review

**DOI:** 10.31083/j.rcm2504145

**Published:** 2024-04-30

**Authors:** Maha Subih, Rami A. Elshatarat, Murad A. Sawalha, Abdulaziz Mofdy Almarwani, Majdi Alhadidi, Mohammad Alrahahleh, Nora H. Elneblawi, Zyad T. Saleh, Raghad Abdelkader, Wesam T. Almagharbeh, Mudathir M. Eltayeb, Nermen A. Mohamed

**Affiliations:** ^1^School of Nursing, Al-Zaytoonah University of Jordan, 11733 Amman, Jordan; ^2^Department of Medical and Surgical Nursing, College of Nursing, Taibah University, 42353 Madinah, Saudi Arabia; ^3^Department of Maternal, Child and Family Health Nursing, Faculty of Nursing, The Hashemite University, 13133 Zarqa, Jordan; ^4^Department of Psychiatric Nursing, College of Nursing, Taibah University, 42353 Madinah, Saudi Arabia; ^5^Department of Nursing, Queen Alia Heart Institute, 11855 Amman, Jordan; ^6^Department of Clinical Nursing, School of Nursing, The University of Jordan, 11942 Amman, Jordan; ^7^Faculty of Nursing, Applied Science Private University, 11937 Amman, Jordan; ^8^Medical Surgical Nursing Department, Faculty of Nursing, University of Tabuk, 47512 Tabuk, Saudi Arabia; ^9^Department of Medical Surgical Nursing, College of Nursing, Prince Sattam Bin Abdulaziz University, 16278 AlKharj, Saudi Arabia

**Keywords:** cardiac rehabilitation programs, coronary artery bypass graft, health-related quality of life, physiological outcomes, postoperative rehabilitation, systematic review

## Abstract

**Background:**

This systematic review explores the impact of cardiac rehabilitation programs (CRPs) on health-related quality of life (HRQoL) and physiological outcomes post-coronary artery bypass graft (CABG) surgery. Acknowledging the increasing importance of CRPs in post-CABG care, the study emphasizes the need for a comprehensive evaluation of their effectiveness. The primary objective is to investigate how CRPs influence HRQoL and physiological outcomes in post-CABG patients, offering insights into the multifaceted impact of these rehabilitation programs.

**Methods:**

A systematic literature review approach was employed to identify relevant studies published between 2013 and 2023. Inclusion criteria encompassed clinical randomized trials and quasi-experimental studies, with a focus on CRP interventions and their impact on HRQoL and physiological parameters.

**Results:**

The review reveals a diverse array of CRP approaches, including exercise training, home-based programs, and telemonitored interventions. Despite methodological variations, a consistent positive impact on HRQoL and physiological outcomes is observed across studies. Noteworthy interventions, such as those incorporating family caregivers, demonstrate holistic benefits. However, limitations include methodological variability and the exclusion of qualitative studies.

**Conclusions:**

This systematic review underscores the substantial positive impact of CRPs on HRQoL and physiological outcomes in post-CABG patients. The diverse approaches and consistent improvements across studies provide a robust foundation for healthcare practitioners and researchers. Future efforts should focus on standardizing CRP interventions and conducting well-designed trials to further enhance the evidence base, facilitating more targeted and effective rehabilitation strategies for CABG patients.

## 1. Introduction

Cardiovascular disease (CVD), particularly those necessitating surgical interventions such as coronary artery bypass grafts (CABG), impose substantial burdens on affected individuals and healthcare systems worldwide. As a critical facet of comprehensive cardiac care, cardiac rehabilitation programs (CRPs) have emerged as integral components in the postoperative management of patients following CABG procedures [1, 2, 3]. These structured interventions encompass a spectrum of exercise, education, and counselling strategies aimed at optimizing patients’ physical, mental, and emotional well-being [3, 4, 5].

This systematic literature review endeavors to explore the multifaceted impact of CRPs on two pivotal aspects—health-related quality of life (HRQoL) and physiological outcomes—in individuals who have undergone CABG. The amalgamation of these components not only seeks to understand the holistic benefits derived from cardiac rehabilitation but also to discern the nuanced interplay between rehabilitation initiatives and the well-being of post-CABG patients [3, 5, 6, 7, 8].

HRQoL serves as a holistic measure that goes beyond conventional clinical endpoints, encompassing subjective experiences, functional capacities, and overall life satisfaction. In contrast, the examination of physiological outcomes provides insights into tangible health markers influenced by cardiac rehabilitation, spanning cardiovascular fitness, exercise capacity, and metabolic and hemodynamic parameters [3, 8, 9].

The rationale for this inquiry stems from the need to augment the existing knowledge base surrounding the efficacy of CRPs specific to CABG patients. By systematically reviewing the existent literature, we aim to identify trends, patterns, and gaps in research, thereby contributing valuable information to guide future interventions, refine existing protocols, and enhance patient outcomes in this critical population [3, 5, 8, 10].

In synthesizing the available evidence, our study aspires to shed light on the intricate relationship between cardiac rehabilitation, HRQoL, and physiological outcomes in CABG patients, fostering a deeper understanding of the potential benefits and areas necessitating further investigation. Ultimately, this exploration holds the potential to inform healthcare practitioners, policymakers, and researchers alike, facilitating the evolution of tailored, evidence-based strategies to optimize the post-CABG care continuum [8, 11, 12].

To comprehensively grasp the influence of CRPs on the HRQoL specifically in patients with CABG, the authors initiated a thorough investigation. Initially, we conducted an extensive search of databases encompassing literature reviews, guidelines, experimental, and quasi-experimental studies published post-2000. The database exploration unearthed a literature review paper considering scientific evidence available up to October 2015, summarizing guidelines from international health organizations and evidence-based practices, along with studies exploring the role of cardiac rehabilitation after CABG [3, 4, 8, 10, 13]. The findings from this literature review and relevant guidelines were synthesized and discussed in the “Background and Overview” section. Regrettably, as of the authors’ knowledge cut-off, no systemic or meta-analysis articles have been published since 2015 specifically delving into the impact of CRPs on HRQoL and physiological outcomes in patients with CABG. Consequently, this study aimed to address the research questions about the significant impact of CRPs on HRQoL and physiological outcomes in CABG patients, as well as the role of cardiac rehabilitation after coronary artery bypass grafting.

Therefore, this study seeks to systematically explore the influence of CRPs on HRQoL and physiological outcomes in individuals post-CABG surgery. Acknowledging the rising significance of CRPs in post-CABG care, a thorough evaluation of their efficacy is crucial. The specific objectives include assessing CRPs’ impact on HRQoL, scrutinizing their effects on physiological outcomes, analyzing literature from 2013 to 2023, and offering insights for healthcare practitioners to better understand CRPs’ role in post-CABG patient care. These objectives aim to focus the study and highlight key investigative areas.

## 2. Background and Overview

### 2.1 History and Background of Cardiac Rehabilitation Post-CABG

The historical development of CABG, initiated by René Favaloro in 1968, has seen methodological innovations such as off-pump surgery and minimally invasive approaches to reduce invasiveness. However, the standard procedure typically involves sternotomy and occasional saphenectomy [8]. CABG is now reserved for patients with complex coronary anatomy and comorbidities, despite advancements, retaining its significance and potential acute-phase complications compared to percutaneous transluminal coronary angioplasty [14, 15]. Additionally, after CABG surgery, common complications such as depression, impatience, physical dysfunction, and isolation may significantly diminish patients’ quality of life [16]. Therefore, evaluating the HRQoL among cardiovascular patients becomes crucial in gauging the impact of cardiovascular and rehabilitation interventions on physical, mental, and social well-being. This assessment serves as a valuable indicator of therapeutic and diagnostic effectiveness [10]. According to Nawito *et al*. [17], HRQoL is a comprehensive evaluation of total well-being, encompassing both physical and social aspects. All facets of life, including work, home, relationships, finances, and health, contribute to the overall assessment of HRQoL [8].

Post-CABG recovery entails a week-long in-hospital stay and a subsequent 2- to 6-week convalescence period. Patients face challenges like heart failure, anemia, atrial fibrillation, and thoracotomy-related pain. Long-term issues include recurrent angina or acute coronary syndrome due to disease progression or bypass failure [8].

Prescribed with a complex drug regimen, post-CABG patients are advised to adopt a healthy lifestyle, encompassing smoking cessation, dietary modifications, exercise, and stress management. Although patients often embrace these behaviors initially, sustaining adherence becomes challenging in the long term. This underscores the multifaceted nature of CABG and highlights the necessity of addressing both immediate and long-term aspects of patient care for comprehensive postoperative management [7, 9].

CRP plays a pivotal role in the early phases following CABG, addressing the critical need for swift physical recovery and the adoption of a lifelong healthy lifestyle and pharmacological regimen [18, 19]. Defined by the British Association for Cardiovascular Prevention and Rehabilitation, cardiac rehabilitation (CR) is described as a coordinated set of activities aimed at positively influencing the root cause of CVD, promoting optimal physical, mental, and social conditions. This definition underscores CR’s role in empowering patients to preserve or resume optimal functioning, potentially slowing or reversing disease progression [18, 19].

CR functions as a two-step comprehensive program tailored to the unique needs of CABG patients. The initial focus is on facilitating a rapid and enhanced recovery from heart surgery during the crucial early weeks post-procedure, particularly significant for the typical CABG patient, often an elderly individual with multiple comorbidities. The second step equips patients with knowledge, tools, and healthy routines essential for successful long-term management of coronary artery disease [8].

### 2.2 Effectiveness of CR on CABG patients: Summarizing Past Literature

Due to the limited availability of recent literature on the topic, including the most recent publications, our primary objective was to conduct a thorough examination of the current literature. We initiated a brief review of international organization guidelines [3, 4, 5, 13] and systematic literature reviews [8], summarizing scientific evidence from 2000 to 2015. This review highlighted the positive impact of CRP on CABG patients, emphasizing improved cardiovascular fitness observed in a study comparing moderate continuous and aerobic interval training. Additionally, CR demonstrated benefits in secondary prevention after acute coronary syndrome, as illustrated by the Global Secondary Prevention Strategies to Limit Event Recurrence After Myocardial Infarction (GOSPEL) study [2]. Major cardiovascular organizations, such as the American Association of Cardiovascular and Pulmonary Rehabilitation and the European Society of Cardiology, underscored the importance of CR in post-CABG care. Divergence in guidelines between 2011 American and 2014 European recommendations reflects ongoing debates in the medical community concerning optimal post-CABG care, considering evolving evidence [3, 13, 20].

This literature review critically evaluated references in the 2011 American College of Cardiology Foundation/American Heart Association guidelines and the 2014 European Society of Cardiology guidelines regarding CR after CABG [3, 13, 20]. Differing classifications for the indication of CR after CABG were noted: class I (level of evidence A) in the former and class II (level of evidence A) in the latter. Despite the lack of recent articles, with most focusing on surrogate endpoints or being over 15 years old, studies post-2006 demonstrated a significant risk reduction in CVD with CR, especially in CABG patients [8]. However, limitations, such as the absence of CABG-only subgroup analysis and varying primary trial quality, were acknowledged. The study emphasized the need for recent and specific research on the impact of CR on post-CABG patients.

Moreover, this literature review critically examined scientific evidence related to CR, emphasizing its benefits in terms of mortality, exercise capacity, quality of life, risk factor control, lifestyle adherence, and return to work, particularly for post-CABG patients. It highlighted two distinct phases in their rehabilitation: the acute phase addressing immediate post-surgery concerns and the maintenance phase promoting the adoption of a lifelong healthy lifestyle. Despite logical expectations based on acute coronary syndrome (ACS) patient outcomes, discrepancies were noted due to the unique challenges of CABG patients. The review underscored the lack of specific multicenter, randomized, controlled trials addressing CR’s impact on CABG-only patients, revealing limitations of observational studies and challenges in conducting randomized controlled trials in the current economic climate [3, 4, 8, 13].

In summary, the current scientific evidence falls short of providing a conclusive role for CR specifically after CABG, despite noted enhancements in quality of life and return to work. The recommendations for post-CABG CR in both US and European guidelines rest largely on extrapolated evidence, lacking validation from dedicated studies [4, 8, 13]. Historical trends caution against assuming logical recommendations as conclusive, emphasizing the need for dedicated research. Advocating for CR as a Class I indication for CABG-only patients is currently untenable, and the launch of Heart Failure [8, 21]: A Controlled Trial Investigating Outcomes of Exercise Training (HF-ACTION) suggests an opportunity for new research [21]. This exploration aims to determine whether a contemporary, comprehensive CR program tailored for CABG-only patients can yield definitive benefits, covering outcomes like mortality, exercise tolerance, and HRQoL over an extended follow-up period. Therefore, we aim to assess recent scientific evidence to support CR for CABG-only patients comprehensively and critically, focusing on experimental studies such as controlled clinical trials and quasi-experimental studies [3, 5, 8].

### 2.3 Significance and Purpose of the Study

This study addresses a critical gap in the existing literature by focusing on the limited understanding of the combined effects of CRPs on both HRQoL and physiological outcomes in individuals who have undergone CABG. Despite the known benefits of CRPs, comprehensive investigations into their dual impact on patients post-CABG remain sparse.

The primary purpose of this systematic review is to consolidate and critically evaluate available evidence from relevant studies, including randomized control trials (RCTs) and quasi-experimental research, to discern the comprehensive impact of CRPs on HRQoL and physiological metrics in the context of CABG patients. By synthesizing findings related to vital signs, anthropometric measurements, blood tests (such as lipid profiles and blood sugar levels), ${\mathrm{VO}}_{2}$peak, muscle endurance, and exercise tolerance, the study aims to provide a holistic perspective on the outcomes associated with CRPs. The research holds significance as it endeavors to inform clinicians, policymakers, and researchers about the potential synergistic benefits of CRPs, offering valuable insights for enhancing post-CABG care protocols and contributing to evidence-based practices in cardiac rehabilitation.

## 3. Methods

To achieve the study’s objectives, a systematic literature review was conducted, focusing on identifying clinical randomized trials and quasi-experimental studies published between 2013 and 2023. This approach served as the designated design and primary methodology.

**Fig. 1.**
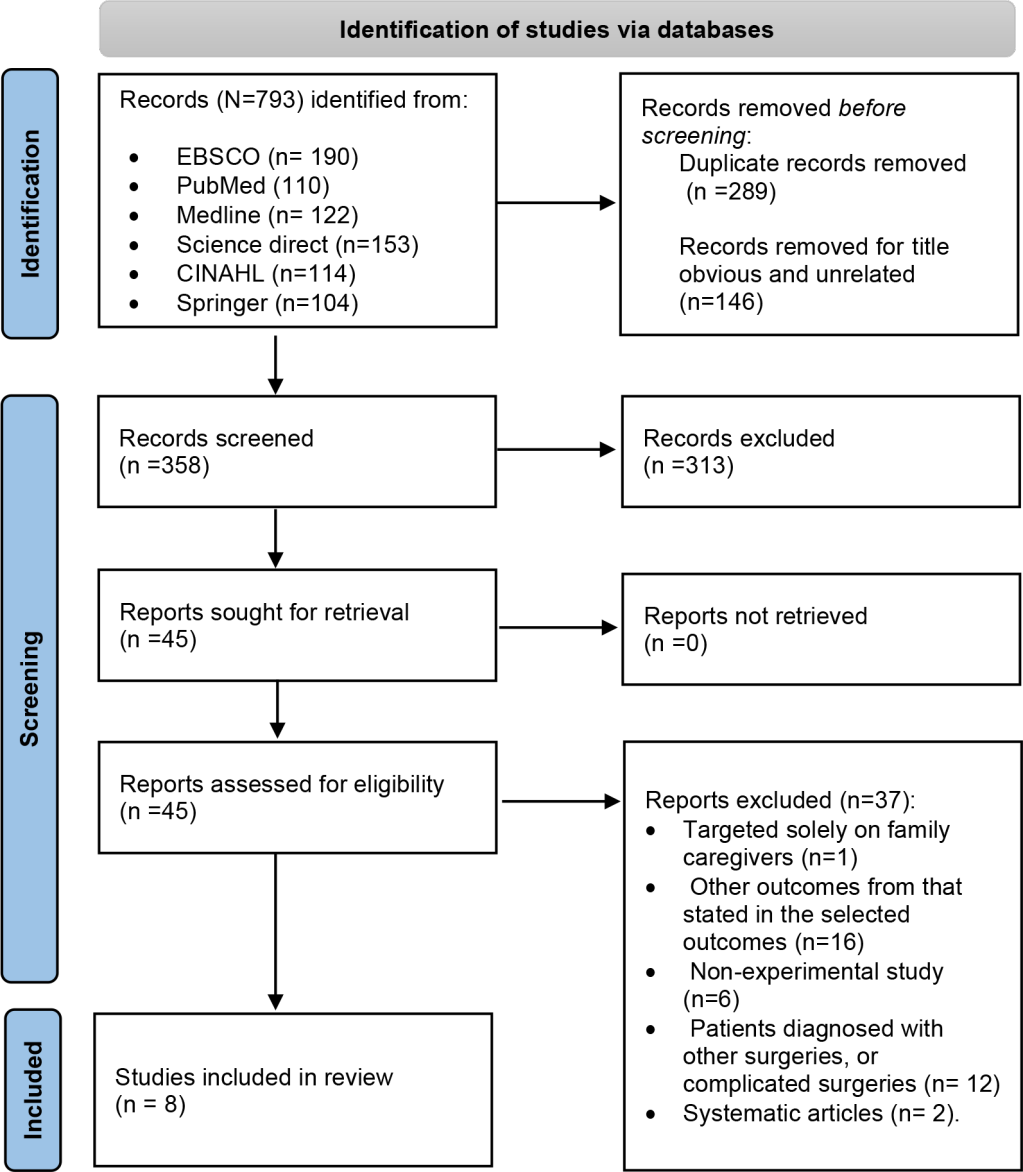
**PRISMA flow chart of database search**. PRISMA, Preferred Reporting Items for Systematic Review and Meta-Analysis Protocols.

The review of recent literature consisted of two stages: the first stage involved searching databases, while the second stage included checking all the related studies found from the first stage to guarantee the precision and consistency of the study. The guideline The Preferred Reporting Items for Systematic Review and Meta-Analysis Protocols (PRISMA) was applied (Fig. 1) [22].

### 3.1 The Inclusion and Exclusion Criteria

The inclusion criteria involved studies of CABG patients, CRPs (excluding educational programs) as the intervention, routine standard care versus standardized programs as comparators, and HRQoL and physiological variables as outcomes.

Exclusion criteria were applied, eliminating studies solely targeting family caregivers, those with outcomes differing from the selected ones, non-experimental studies, patients undergoing other surgeries or complicated surgeries, and systematic articles.

### 3.2 Search Strategy and Data Sources

Regarding the search strategy, PICO-related terms were employed, emphasizing CABG patients as the population, CRP (excluding educational programs) as the intervention, routine standard care versus standardized programs as comparators, and HRQoL and physiological variables as outcomes. The search spanned PubMed, EBSCO Host, Springer Link, Science Direct, CINAHL, and Medline from 2013 to 2023, utilizing specific keywords such as “Coronary artery bypass graft”, “CABG”, “Cardiac rehabilitation program”, “Cardiac rehabilitation intervention”, “Health-related quality of life”, “Randomized control trial”, “Experimental”, and “Quasi-experimental”. Additionally, inclusion and exclusion criteria were applied, restricting the search to adult populations, English language, and inpatient and outpatient settings, while excluding articles involving educational programs or non-experimental studies.

### 3.3 Quality Assessment of Selected Studies

For quality assessment, two cardiologist physicians independently reviewed titles and abstracts to identify potentially relevant studies. Full texts were obtained for these studies, and methodological quality was assessed using the CONSORT checklist for RCTs [23] and the Transparent Reporting of Evaluations with Nonrandomized Designs (TREND) checklist for non-RCTs [1]. CONSORT, with 25 items, appraised RCTs’ methodological quality, and TREND, with 22 items, assessed non-RCTs’ quality [23]. Each matching item contributed a point, resulting in CONSORT scores ranging from 0 (low quality) to 25 (high quality) and TREND scores from 1 to 22 [1].

### 3.4 Data Extraction

Data extraction involved specific details about populations, settings, methods, and interventions. Two physicians independently performed data extraction, resolving any discrepancies through consultation with a third author.

The literature search initially identified 793 studies, reduced to 358 after removing duplicates. Screening of titles and abstracts resulted in 45 studies related to CABG patients. Exclusion criteria focused on psychological interventions, refining the selection to eight eligible studies. The PRISMA 2020 flow diagram illustrates the study selection process, ultimately leading to the inclusion of eight studies in the literature review [22].

## 4. Results

In the meticulous process of study selection, we rigorously applied exclusion criteria to refine the scope of our investigation. Out of 37 reports, one was excluded as it exclusively focused on family caregivers, and 16 were dismissed due to presenting outcomes beyond those specified in our selected criteria. Additionally, six non-experimental studies were excluded, along with 12 studies involving patients diagnosed with other surgeries or complicated procedures and two systematic articles. The application of these stringent criteria culminated in the inclusion of eight studies for our systematic review. The following section provides a detailed exploration of the outcomes and findings gleaned from these carefully selected studies, contributing valuable insights into the impact of CRPs on HRQoL and physiological outcomes in post-CABG surgery patients.

The summarized review of studies presented in Table 1 (Ref. [6, 11, 12, 16, 24, 25, 26, 27]) encompasses four Randomized Control Trials (RCTs) and four quasi-experimental studies, examining the impact of CRPs on HRQoL and physiological outcomes in CABG patients. Both inpatient and outpatient settings were considered in the final assessment. Across these studies, a consistent positive effect of CRPs on HRQoL and physiological variables was observed.

**Table 1.** **Summary of the included studies**.

No	Study/Setting	Sample Size	Interventions	Outcome Measures	Results
1.	Zafari Nobari *et al*. [12], 2021/Iran	CEG = 48 HLEPG = 49	-The HLEP was implemented in four (45–60-min) training sessions held in 3–5 groups and face-to-face training and sharing experiences among patients. The contents of HPBs. The content was compiled into an educational booklet and a pamphlet. Telephonic monitoring was performed in the 4th & 8th weeks after discharge. -The participants of CEG received conventional education at discharge time. Include a low salt diet, 30-min daily walk, and medication adherence. Performed at the bedside and lasted 5–10 min on the discharge day.	-HRQoL -Adherence to HPB	-Both groups showed a significant increase in the mean score of HRQoL but this increase was visibly greater in the HLEPG. -The healthy lifestyle empowerment program also significantly increased the mean score of adherences to HPB in the HLEPG, whereas no such change was observed in the CEG.
2.	Zolfaghari *et al*. [16], 2018/Iran	Control group = 25 PT group = 25	-The patients in the PT group underwent 16 sessions of 15‐min PT in total: four sessions of PT 2–5 days after surgery and 12 sessions thereafter (3 times a week for 1 month). -The techniques included positioning and postural drainage, chest tapotement, coughing exercises, breathing exercises, and thorax mobilization exercises. -The control group received the standard post‐CABG surgery care; and completed four 15‐min sessions in the 2–5 days immediately following surgery.	-Short-form 36 (SF-36) measures QoL (physical and mental component)	-The QoL scores of the PT group significantly improved after the intervention. A significant difference between groups was observed in both the physical and mental component summary.
3.	Alexiev *et al*. [6], 2017/Bulgaria	ICR group = 50 RCR Group = 50	-RCR-program consists of early mobilization, breathing exercises, pulmonary clearing techniques, range of motion exercises, psychological counselling and risk factors management along with optimal pharmacological treatment. Hemodynamics monitoring was obtained during tests. -ICR-program sessions, additional strength or flexibility exercises were included, according to the patient’s needs.	-Anthropometric measurements (including height, weight, Body-Mass Index, waist, hip, and chest circumference) was obtained -Vital signs -Systolic and Diastolic Blood Pressures and Resting Heart Rate -Basic laboratory results (full blood count, serum lipids level, creatinine and glucose levels), ECGs, Holter-ECGs, pre- and post-operative radiographs -Euro QoL -6-MWT	-ICR-group did better than RCR-group in the 6-MWT. After significant difference in walked distance between CABG and valve surgery patients and in men compared to women was found. -Gender, age, comorbidities, and type of surgery were independently associated with the level of functional capacity improvement at discharge.
4.	Salavati *et al*. [26], 2016/Iran	Control group = 30 Interventional group = 30	-The interventional group received home-based cardiac rehabilitation programs includes: information about their disease, usual signs and symptoms and potential complications of their illness, prescribed medications, potential change in their lifestyle which they have to know in order to go to the hospital on time, also received one simplified booklet about their illness. instructed in the training program (four sessions a week in the hospital (and 3 days left, at home based on the training, is given at hospital) for 5 weeks and a total of 20 sessions) and guide to the optimal training effort. In between the visits (three home visit includes: days 7, 27 and 47 after discharge). -The Control group received usual educational care.	-MacNew Heart Disease HRQoL	-At the time of pretest, an insignificant difference was found in the mean score of HRQoL between the two groups. -Although the mean score of HRQoL in all patients in both groups increased two months after the intervention, this increase in patients in the interventional group was statistically higher.
5.	Pačarić *et al*. [25], 2020/Croatia	One group = 47	-Rehabilitation program -The content of the program not specified.	-SF-36 -SF-12 -SF-6D	-After rehabilitation, there was a significant improvement in all domains of quality of life. The highest score was change in pain; the lowest quality after rehabilitation was limitations due to physical difficulties; patients with coronary heart disease have a poor quality of life before surgery. -One month after the surgery, the quality of life improved but was still inadequate. -One year after surgery, satisfactory results were obtained in almost all subscales.
6.	Akbari and Celik [11], 2018/Iran	Control group = 50 Intervention group = 50	-An educational booklet was provided and the discharge plan provided with care‐related training in small three‐person groups using teaching methods such as lecture, question‐and‐ answer, demonstration, feedback‐giving, reinforcement, and summarization. -Training sessions held before and after CABG. -Sessions lasted 60–220 min with a mean of 140 min, at least one family member of each patient attended the sessions. -The educational booklet was developed based on disease, procedure, and complications of CABG, prevention of the coronary artery blockage, and post‐CABG self‐care activities. -Counselling was provided via both home visits and follow‐up telephone contacts at 2 and 10 days and 6 weeks after discharge.	-SF‐36	-The baseline mean scores of QoL in the control and the intervention groups were with no difference. -Six weeks after hospital discharge, the mean score of QoL in the intervention group was significantly greater than the control group.
7.	Laustsen *et al*. [24], 2020/Denmark	One group = 34	-TCR Intervention patients were trained 2–6 weeks after hospital discharge, three times weekly for 12 weeks. -At the first consultation, participants received basic information on how exercise impacts their disease and health. -Participants set their own goals and choose their own exercise mode i.e., running, road biking, spinning or going to the local fitness centre.-Participants underwent a cardiopulmonary exercise test (${\mathrm{VO}}_{2}$peak). -Muscle endurance was measured during this test. Participants were encouraged to exercise for at least 60 min, with 20 min of moderate to high-intensity exercise in each session. -Participants were instructed to exercise for 20 min per session with a moderate intensity between 40–60% of HR reserve during the first four weeks, and for 20 min per session with a high the intensity of 60–84% of HR reserve during the last eight weeks. -Muscle power and muscle strength were tested. -Three experienced physiotherapists gave individual weekly feedback on exercise training intensity by e-mail, Skype, phone or short message service (SMS) according to patient preferences. -After ending TCR, participants were encouraged to continue the exercise without the monitoring equipment. -Conventional CR was provided three times weekly for 12 weeks, consisting of group-based exercise training supervised by hospital physiotherapists on cardio-protective lifestyles.	-${\mathrm{VO}}_{2}$peak: changes in physical capacity and muscle endurance -Muscle powerM -HRQoL (SF-36)	-A significant increase in peak oxygen uptake of 10%, in muscle endurance of 17%, in muscle power of 7%, and in muscle strength of 10% after the telemonitored exercise-based cardiac rehabilitation program. -HRQoL was significantly improved by 19% in the physical and 17% in the mental component scores. -No significant improvement in peak oxygen uptake between baseline and 12 months follow-up, but a significant improvement in muscle endurance (0.3 watts/kg), muscle power (0.4 watts/kg; 0.2–0.5), muscle strength (0.5 N/m/kg; 0.1–0.9).
8.	Spiroski *et al*. [27], 2017/Serbia	One group = 54	-The inpatient program; was implemented 7 times a week for a period of 3 weeks. It’s included exercise training, information sessions, dietary counselling, psychosocial support, and smoking cessation. -There were 2 training sessions daily, each of 45 minutes duration. The first training session which included a warm-up and cool-down period and a 30-minute training phase (aerobic interval training consisting of 3 minutes of exercise and 3 minutes of rest on a cycle ergometer). -The second session include aerobic training included walking on a flat surface and walking upstairs. -The outpatient program was implemented 5 times a week for a period of 6 months.	-Exercise tolerance -Peak respiratory exchange ratio -Peak ${\mathrm{VO}}_{2}$ -Peak ${\mathrm{VCO}}_{2}$ -Peak ventilatory exchange -Peak breathing reserve	-After 3 weeks of an exercise-based cardiac rehabilitation program, exercise tolerance improved as compared to baseline, as well as peak respiratory exchange ratio. Peak ${\mathrm{VO}}_{2}$, peak ${\mathrm{VCO}}_{2}$, peak ventilatory exchange and peak breathing reserve were also improved. -Improvement trend continued after 6 months. -No major adverse cardiac events were noted during the rehabilitation program.

CEG, conventional education group; HLEPG, healthy lifestyle empowerment program group; HPB, health-promoting behaviors; PT, physiotherapy; CR, cardiac rehabilitation; ICR, individualized cardiac rehabilitation group; RCR, routine control group; TCR, telemonitored exercise-based cardiac rehabilitation; HRQoL, health related quality of life; SF, short-form; QoL, quality of Life; CABG, coronary artery bypass graft; 6-MWT, 6-minute walk test; ${\mathrm{VO}}_{2}$, maximal oxygen consumption; ${\mathrm{VCO}}_{2}$, maximum carbon dioxide consumption; HLEP, healthy lifestyle empowerment program; ECG, electrocardiogram; HR, heart rate.

The review encompassed studies focusing on inpatients [6, 11, 12, 16] and outpatients [24], with some studies considering both [25, 26, 27]. Every study confirmed the beneficial influence of CRPs on both HRQoL and physiological outcomes in CABG patients. The methodological quality exhibited variability, with Zafari Nobari *et al*. [12] (2021) receiving a high rating, while others ranged from moderately high to low moderate. The effect of CRPs on HRQoL was consistently positive across studies. Moreover Zafari Nobari *et al*. [12] (2021) demonstrated superior HRQoL improvement with a healthy lifestyle program. Zolfaghari *et al*.’s [16] physiotherapy (PT) group exhibited significant mental and physical HRQoL improvements. Salavati *et al*. [26] (2016) found a significant HRQoL enhancement with a home-based CRP. Akbari and Celik [11] (2018) noted effective HRQoL improvement with discharge training and postoperative counselling. Three studies reported significant increases in mental and physical HRQoL [24, 25, 27]. The reviewed studies collectively underscored the positive impact of CRPs on HRQoL and physiological outcomes in CABG patients.

Cardiac rehabilitation guidelines recommend diverse exercises for post-CABG patients, spanning anaerobic, resistance, and flexibility training. Intensity ranges from 40–85% resting heart rate, lasting 20–60 minutes, three to five times weekly for 6 to 12 weeks. Zafari Nobari *et al*.’s study [12] employed a health-promoting lifestyle program (HELP) in four sessions, addressing nutrition, physical activity, spirituality, relations, health responsibility, and stress management. Salavati *et al*. [26] utilized usual education and home-based CRPs, covering disease education, signs, complications, drug treatment, lifestyle changes, and workouts [12]. Laustsen *et al*. [24] implemented a 12-week tele-monitored exercise-based CRP. Various interventions, including counselling, physiotherapy, and psychological support, demonstrated improvements in mental and physical health, emphasizing the need for individualized programs [24].

In detail, the following presents the reports on the methodological approaches and results of the Randomized Control Trials (RCTs) or quasi-experimental studies that explored the effectiveness of CRPs on HRQoL, as identified and listed in Table 1.

In the study conducted by Zafari Nobari *et al*. [12] (2021) in Iran, a total of 97 CABG patients were divided into two groups: the Healthy Lifestyle Empowerment Program Group (HLEPG) with 49 participants and the Conventional Education Group (CEG) with 48 participants. The HLEPG received a health-promoting lifestyle program (HLP) delivered in four training sessions, incorporating face-to-face interactions and shared experiences. The HLP content was compiled into an educational booklet and pamphlet, and telephonic monitoring was conducted in the 4th and 8th weeks post-discharge. The CEG received conventional education at discharge, including a low-salt diet, a 30-minute daily walk, and medication adherence. Both groups exhibited a significant increase in HRQoL mean scores, with a visibly greater improvement in the HLEPG. Additionally, the HLEPG showed a significant increase in adherence to health-promoting behaviors compared to the CEG [12].

In Zolfaghari *et al*.’s study [16] (2018) in Iran, 50 patients who underwent CABG surgery were randomly assigned to a control group (n = 25) and a physiotherapy (PT) group (n = 25). The PT group underwent 16 sessions of 15-minute PT, incorporating various techniques such as positioning, postural drainage, chest tapotement, coughing exercises, breathing exercises, and thorax mobilization exercises. The control group received standard post-CABG surgery care. The Short-Form 36 (SF-36) was used to measure QoL in both the physical and mental components. The PT group exhibited a significant improvement in QoL scores compared to the control group [16].

Alexiev *et al*.’s study [6] (2017) in Bulgaria included 100 patients divided into two groups: Individualized Cardiac Rehabilitation (ICR) group (n = 50) and Routine Control Rehabilitation (RCR) group (n = 50). The ICR program consisted of early mobilization, breathing exercises, pulmonary clearing techniques, range of motion exercises, psychological counselling, and risk factor management. The RCR program included group-based exercise training supervised by hospital physiotherapists. Outcome measures included anthropometric measurements, vital signs, basic laboratory results, Euro QoL, and the 6-minute walk test (6-MWT). The ICR group performed better in the 6-MWT, and factors such as gender, age, comorbidities, and type of surgery were independently associated with the level of functional capacity improvement at discharge [6].

Salavati *et al*.’s study [26] (2016) in Iran compared a control group (n = 30) with an interventional group (n = 30) in terms of HRQoL using the MacNew Heart Disease HRQoL questionnaire. The interventional group received home-based CRPs, including information about the disease, signs and symptoms, potential complications, prescribed medications, lifestyle changes, and workout programs. The control group received usual educational care. Although both groups showed an increase in HRQoL two months after the intervention, the interventional group exhibited a statistically higher increase [26].

Pačarić et al.’s study [25] (2020) in Croatia involved 47 patients undergoing cardiac rehabilitation after surgery. The rehabilitation program significantly improved all domains of quality of life, with the highest improvement in pain and the lowest in limitations due to physical difficulties. The study highlighted the importance of rehabilitation in enhancing the quality of life for patients with coronary heart disease [25].

Akbari’s study [11] (2018) in Iran included 100 patients undergoing CABG surgery, divided into a control group (n = 50) and an intervention group (n = 50). The intervention group received an educational booklet, discharge training, and post-discharge counselling. The control group received routine discharge and post-operative instructions. The Short-Form 36 (SF-36) was used to measure QoL. The baseline mean scores of QoL were similar between the groups, but six weeks after hospital discharge, the intervention group showed a significantly greater mean score of QoL compared to the control group [11].

Laustsen *et al*.’s study [24] (2020) in Denmark included 34 participants in a tele-monitored exercise-based cardiac rehabilitation program (TCR) conducted 2–6 weeks after hospital discharge, three times weekly for 12 weeks. The program resulted in a significant increase in peak oxygen uptake, muscle endurance, muscle power, and muscle strength. HRQoL measured using the SF-36 showed a significant improvement in both the physical and mental component scores [24].

Spiroski *et al*.’s study (2017) [27] in Serbia involved 54 patients after CABG surgery. The inpatient program included exercise training, information sessions, dietary counselling, psychosocial support, and smoking cessation for three weeks. The outpatient program continued for six months. The study measured exercise tolerance, peak respiratory exchange ratio, peak ${\mathrm{VO}}_{2}$, peak ${\mathrm{VCO}}_{2}$, peak ventilatory exchange, and peak breathing reserve. After the exercise-based cardiac rehabilitation program, there was an improvement in exercise tolerance, peak respiratory exchange ratio, peak ${\mathrm{VO}}_{2}$, peak ${\mathrm{VCO}}_{2}$, peak ventilatory exchange, and peak breathing reserve. The improvement trend continued after six months, and no major adverse cardiac events were noted during the rehabilitation program [27].

Together, these studies emphasize the favorable influence of diverse CRP on the QoL and functional capacity of individuals undergoing coronary artery bypass graft surgery. Various interventions, ranging from lifestyle empowerment programs to physiotherapy and telemonitored exercise-based programs, exhibited noteworthy enhancements in both the physical and mental aspects of HRQoL. These findings underscore the significance of personalized rehabilitation approaches in optimizing outcomes for post-CABG surgery patients.

## 5. Discussion

This systematic review illuminates the pivotal role of CRPs in enhancing both HRQoL and physiological outcomes in patients undergoing CABG. Drawing on a mix of inpatient and outpatient interventions, the selected studies consistently highlight a positive influence, offering vital insights for clinical practice and shaping the trajectory of future research.

The comprehensive evaluation of diverse CRPs unravels a spectrum of approaches, including exercise training, home-based programs, telemonitored interventions, and personalized physiotherapy [12, 16, 27]. This diversity underscores the complexity of health-promoting behaviours and delivery methods employed in CRPs, ranging from face-to-face training to educational booklets, lectures, and home visits.

CRPs consistently exhibit a positive effect on both HRQoL and physiological variables, with variations in session frequencies and durations. Sessions ranged from four lasting 45–60 minutes [12, 26], three times a week for 12 weeks [24], 60–220-minute sessions [11], 16 sessions of 15 minutes each [16], to seven times a week for three weeks for inpatients and five times a week for six months for outpatients [27].

Despite methodological heterogeneity, a common theme emerges—a substantial enhancement in HRQoL for CABG patients engaged in CRPs. Interventions like Zafari Nobari *et al*.’s [12] Health Lifestyle Empowerment Program and Zolfaghari *et al*.’s [16] physiotherapy sessions specifically underscore improvements in both mental and physical HRQoL components.

Positive trends extend to physiological outcomes, encompassing vital signs, exercise capacity, and biochemical parameters. Studies by Spiroski *et al*. [27] and Pačarić *et al*. [25] (2020) highlight significant improvements in heart rate, blood pressure, blood sugar, and lipid profiles post-CRP, emphasizing the holistic benefits of these rehabilitation programs.

The variability in methodologies and quality assessments adds richness to the evidence presented. While Zafari Nobari *et al*. [12] (2021) and Zolfaghari *et al*. [16] (2018) achieved high-quality ratings, others ranged from moderately high to low moderate. This variability underscores the imperative of standardized reporting and methodological rigor in future research, ensuring comparability across studies.

The positive correlation observed between CRPs and improved HRQoL and physiological parameters holds particular relevance for CABG patients navigating recovery challenges. The multifaceted nature of CRPs, incorporating education, exercise, and psychosocial support, aligns with the comprehensive needs of this patient population [11, 12, 26].

While acknowledging overall positive trends, the discussion illuminates potential limitations. Heterogeneity in interventions, outcome measures, and follow-up durations introduces variability in results. Additionally, the moderate methodological quality of some studies underscores the need for more high-quality randomized controlled trials in this domain [6, 16, 25].

In summary, this systematic review underscores the consistently positive impact of CRPs on HRQoL and physiological outcomes in post-CABG patients. The diverse approaches explored contribute to a robust foundation for healthcare practitioners and researchers. Despite intervention variations, the overall trend indicates significant improvements in post-CABG patients, highlighting the effectiveness of rehabilitation efforts. The study recommends future standardization of CRP interventions and well-designed trials to enhance the evidence base. This collective evidence reinforces the crucial role of tailored rehabilitation in enhancing the well-being of post-CABG individuals. On the contrary, a prior literature review’s conclusion regarding CRP after CABG recognizes the advantages of surrogate endpoints but raises concerns about the absence of clear evidence supporting reduced mortality. The reservations about categorizing CR as a Class I recommendation for CABG-only patients underscore the need for new research initiatives. The proposal advocates for a comprehensive CR program, challenging the CR community to provide unequivocal evidence for its efficacy in this specific patient population over an extended follow-up period.

### 5.1 Study Limitations

This study, exploring the impact of CRPs on HRQoL and physiological outcomes in post-CABG patients, acknowledges several limitations. The investigation encompassed diverse CRP application approaches, leading to variations in findings, making it challenging to establish a standardized framework. The exclusion of qualitative studies limits insights into patient perspectives, experiences, and perceptions of CRPs, hindering a comprehensive understanding beyond quantitative outcomes. Recognizing the need for an integrative systematic review, combining quantitative and qualitative evidence, is crucial for a nuanced understanding of CRPs’ effectiveness and feasibility. Potential publication bias, selective reporting, a knowledge cut-off date, and heterogeneity in study designs introduce complexities, necessitating cautious interpretation. Addressing these limitations in future research will enhance comprehension and inform effective CRP implementation strategies for post-CABG patients.

### 5.2 Study Implications and Recommendations

Healthcare practitioners are urged to tailor CRPs to individual patient needs, incorporating a range of health-promoting behaviors for optimal engagement and outcomes. The involvement of a multidisciplinary team, including nurses, physiotherapists, and dietitians, underscores the versatility of rehabilitation interventions and emphasizes the significance of collaborative support.

Integrating family caregivers into CRPs proves beneficial, emphasizing collaborative self-care activities and enhancing overall support for post-CABG patients. Standardizing the reporting of CRP interventions and methodologies is crucial for enhancing comparability across studies, fostering a comprehensive understanding of the effectiveness of diverse approaches.

For future research, longitudinal studies are recommended to gain insights into the sustained effects of CRPs, guiding the development of extended and targeted rehabilitation programs. Incorporating qualitative methodologies can offer a holistic understanding of patient experiences and perceptions, complementing quantitative data.

Economic evaluations are essential to address the identified limitation of insufficient data, providing valuable insights into the cost-effectiveness of CRPs. Prioritizing well-designed RCTs will contribute to advancing the evidence base and refining the classification of different rehabilitation modes based on their analyzed outcomes.

Additionally, healthcare practitioners are advised to implement continuous monitoring and evaluation mechanisms within CRPs, allowing for real-time adjustments to interventions based on individual patient progress and feedback. Developing personalized care plans, considering the unique needs and preferences of patients, is crucial to fostering a patient-centered approach in CRPs.

Furthermore, the establishment of standardized outcome measures for assessing HRQoL and physiological parameters will enhance the consistency and comparability of research findings across various studies. Collaborative efforts among healthcare institutions and researchers to establish a centralized database for CRP outcomes can facilitate meta-analyses and contribute to a more comprehensive understanding of the long-term effects of these programs. Lastly, fostering patient education and awareness regarding the benefits and importance of participating in CRPs is essential for increasing adherence and promoting positive health behaviors.

## 6. Conclusions

This review delved into the quality of studies investigating the influence of CRPs on HRQoL for CABG patients. The collective evidence from these studies consistently affirmed a positive impact on HRQoL for both inpatients and outpatients post-CABG surgery. However, CRPs exhibited variations among studies, lacking comprehensive coverage of essential aspects for CABG patients, and displaying differences in intervention timing. Notably, a focus on inpatients dominated the literature, highlighting the need for more research directed at outpatient populations. Furthermore, the synthesis of reviewed studies revealed that CRPs combining physical exercise, dietary interventions, and educational components significantly influenced physiological outcomes, including heart rate, cholesterol levels, triglycerides, and body mass index. This underscores the importance of refining strategies to seamlessly integrate CRPs into standard treatment protocols for CABG patients, emphasizing the potential for comprehensive interventions to enhance both HRQoL and physiological parameters.

## Supplementary Material

Supplementary material associated with this article can be found, in the online version, at https://doi.org/10.31083/j.rcm2504145.
